# Characteristics of the salivary microbiota in cheilitis granulomatosa

**DOI:** 10.4317/medoral.23041

**Published:** 2019-10-27

**Authors:** Yang Liu, Qian Zhang, Xiaosheng Hu, Feng Chen, Hong Hua

**Affiliations:** 1 Department of Oral Medicine, Peking University School and Hospital of Stomatology & National Engineering Laboratory for Digital and Material Technology of Stomatology & Beijing Key Laboratory of Digital Stomatology, Beijing, China; 2 Central Laboratory, Peking University School and Hospital of Stomatology & National Engineering Laboratory for Digital and Material Technology of Stomatology & Beijing Key Laboratory of Digital Stomatology, Beijing, China

## Abstract

**Background:**

Cheilitis granulomatosa (CG) is a disturbing and persistent idiopathic lip swelling. The cause and treatment has not been wholly elucidated. Some reports infer that CG is mainly associated with dental infection but no firm or reliable microbiological evidence has been provided for a causative organism. This study aimed to evaluate whether microorganisms contribute to the etiology of CG in order to inform appropriate treatment options in clinic.

**Material and Methods:**

Unstimulated saliva was collected from 15 CG patients who were diagnosed clinically and pathologically and 15 healthy controls (HC). DNA was extracted from the precipitate of the centrifuged saliva for 16s rRNA high-throughput sequencing using the Miseq PE300 platform. The distribution of the microbiome between the two groups was compared.

**Results:**

CG patients had a greater microbial flora that was more diverse than the HC. *Prevotella*, *Alloprevotella*, *Porphyromonas*, *Actinomyces*, *Rothia*, *Fusobacterium*, *Haemophilus*, and *Aggregatibacter* had a significantly higher abundance in CG patients. In contrast, *Streptococcus* and *Campylobacter* were the most abundant genera in HC with a mean relative abundance of 63% and 2%, respectively. The microbiological network indicated that most of the bacteria that were enriched at greater levels in CG patients were likely to be *Prevotella*, *Actinomyces*, and *Rothia*. These have been shown to co-exist with other bacteria.

**Conclusions:**

The composition and structure of bacterial communities in CG patients were different from HC. Most of the genera observed in CG patients were associated with periodontitis and pulp infection. These findings might be helpful in understanding the etiology of CG. Further study will be needed to confirm these findings and explore the underlying pathological mechanism.

** Key words:**Cheilitis granulomatosa, 16s rRNA high-throughput sequencing, salivary microbiota.

## Introduction

First described by Miescher in 1945 (1), Cheilitis granulomatosa (CG) is a rare disease presenting as a persistent, idiopathic swelling of the lip in the absence of systemic disease such as Crohn’s disease and sarcoidosis. The incidence of CG has been estimated at 0.08% in the general population and there appears to be no specific predisposition by ethnicity, sex, or age (2,3). The etiology of CG has not been clearly elucidated, although proposed causes include chronic dental inflammation, focal infections, metal allergy from dental crowns, dietary allergens such as cinnamon, benzoates, and hereditary predisposition. However, the precise cause remains unknown(4). Options for treatment include dietary modifications, antibiotics, systemic or intralesional corticosteroids and surgery but no definitive treatment has been recommended because of the poorly understood mechanisms (3).

The importance of odontogenic infection in the development of CG has been reported in recent several decades. Elimination of dental infectious foci resulted in regression or disappearance of swelling in 11 out of 16 patients (5). Furthermore, other studies also proved that the swelling of the lip improved greatly after the treatment of apical periodontitis without any drugs (6,7). Consequently, it could be inferred that CG is associated with dental infection but no further reliable evidence has been provided for a causative microorganism. To date, no study has addressed the microbial composition and structure of oral flora in CG patients.

Traditional bacterial culture and identification is both time- and labor-consuming, while detection of some anaerobic bacteria can be difficult to isolate and identify. High-throughput sequencing technology of 16S rRNA offers the advantage of a larger number of sequences being resolved with a higher discrimination, which aids large-scale analysis and characterization of the human bacterial community. This technique has been applied for the study of oral diseases such as periodontitis (8), dental caries (9,10), and halitosis (11). Saliva and plaque are commonly used as representative samples for analysis. Therefore, we aimed to investigate the microbiome composition of saliva samples from patients with CG and explore whether there were specific microorganisms that would be different from healthy subjects.

## Materials and Methods

- Participant recruitment 

A case-control study was conducted, using the Strengthening the Reporting of Observational Studies in Epidemiology (STROBE) guidelines for reporting the methods and results. From January 2013 to June 2014, a total of 15 CG patients (7 men, 8 women; average age 47.6 ±3.3 years) and 15 healthy controls (HC) (5 men, 10 women; average age 53.7±1.7 years) were recruited from Peking University School and Hospital of Stomatology. The inclusion criteria of CG patients: 1. Typical clinical manifestations of persistent non-tender, diffuse, firm swelling of the upper lip, lower lip or both; 2. Pathological diagnosis was confirmed by pathological specialist based on the criteria of non-caseous granulomatous inflammation in the deeper subcutaneous and parafollicular tissues or intercellular swelling(4). Exclusions of CG patients: The swelling of lip caused by Crohn’s disease, sarcoidosis, tuberculosis and other systemic diseases. The HCs were recruited at the same period matched with CG patients. All the CG patients and controls in our study were not treated by topical or systemic antibiotics and corticosteroids in the previous three months before the saliva collection. All participants were examined for oral hygiene, using the technique of probing depth (PD) and decayed-missing-filled-teeth (DMFT). After the inquiry and detailed clinical examination, X-ray was taken for all the suspicions teeth. The following information was collected and recorded: age, gender, PD, DMFT, duration, lower or upper lip.

The study protocol was reviewed and approved by the Ethics Committee of Peking University School and Hospital of Stomatology (PKUSSIRB-201412007). All participants received both written and oral information before they gave their informed consent to participate in the study.

- Sample collection and DNA extraction

All participants were not allowed to eat or drink an hour before saliva collection. They washed their mouths with pure water only. 1ml unstimulated saliva were spitted into a 50ml sterile tube, placed in ice and delivered to laboratory, then stored immediately at -80ºC for further analysis.

The saliva samples were centrifuged at 6010 g for 15 min and the precipitate was collected for DNA extraction. The precipitate was spitted by Lysozyme (20 mg/mL, 37ºC for 1 h), which was used to obtain better yields of difficult to lyse gram positive bacteria. Then, the total bacterial genomic DNA was extracted using a QI*Aa*mp DNA Mini Kit (Qiagen, Valencia, CA, USA) by the protocol according to the manufacturer’s instructions. The quality and quantification were identified by 1% agarose gel and a Qubit Fluorometer (Invitrogen, USA). The high quality DNA with the OD260/OD280=1.8-2.0, concentration higher 50ng/ul was selected to sequence.

- Miseq Sequencing 

The V3-V4 region of 16S rRNA gene was sequenced using the Illumina Miseq pair-end method. The primers were: 338F 5’-ACTCCTACGGGAGGCAGCA-3’ and 806R 5’-GGACTACHVGGGTWTCTAAT-3’. Unique eight base barcode sequences were added adjoining the primer at the 5’ position to distinguish between different samples. The concentration of average sample as PCR template was diluted to 10ng/ul. The adapter sequences and sample-specific barcode sequences were amplified by the following program: initial denaturation for 5 min followed by 25 cycles at 95ºC for 30 s; 55ºC for 30 s; and 72ºC for 30 s. This was then followed by an extension step at 72ºC for 10 min and a cooling step at 4ºC. The PCR products were tested by 2% agarose gel electrophoresis and purified using Agencourt AMPure XP (Beckman Coulter, Inc., CA, USA). The integrity and concentration of samples was tested with an Agilent 2100 bioanalyzer (Agilent Technology, USA) and real-time RCR, which were performed with an ABI7500 Real-time PCR System (Applied Biosystems, USA). The amplicons were then pooled together for sequencing. The sequencing data were submitted to Short Reads Archive database with the accession number was SRP157005.

- Sequencing process

Sequences were trimmed with the Q30 value from the raw data. High quality sequences of base length >300 bp, primer mismatch <2 and barcode without mismatch were used for further analysis. Chimeric sequences were removed using the Usearch software version 7.0 software and QIIME. The high quality sequences were blasted with the Ribosomal Database Project (RDP) (12) using MOTHUR (version 1.30.1) (13) and clustered into operational taxonomic units (OTUs) at the 97% similarity using UPARSE software. Then, alpha diversity (Chao 1, observed OTUs, PD whole tree, Shannon) and Principal  co-ordinates  Analysis (PCoA) was drawn with MOTHUR to compare the differences in salivary microbiota structure for CG and HC individuals. Co-occurrence networks were generated using Cytoscape software (version 3.3.1).

- Statistical Analyses

Independent t-test analysis was used to compare the age and PD; alpha diversity (Chao, observed OTUs, PD whole tree and Shannon) was analyzed by Kruskal-Wallis test; chi-square was used to analyze gender between two groups. Differences in relative abundance of microbial communities in HC and CG individuals were compared using the Wilcoxon rank-sum test. P-values <0.05 were considered to be significant differences between the two groups. We calculated the Pearson correlation coefficients (PCC) for all the OTUs and used the permutation test to compare the statistical significance of the PCC value (*P*<0.01 was set for significance). The pairwise permutational multivariate analysis of variance (PERMANOVA), using the Adonis analysis, was conducted to compare the significant difference of beta-diversity.

## Results

- Basic sequencing information of CG and HC subjects

There was no significant difference in sex, age and oral hygiene between the two groups (Table 1).

**Table 1:**
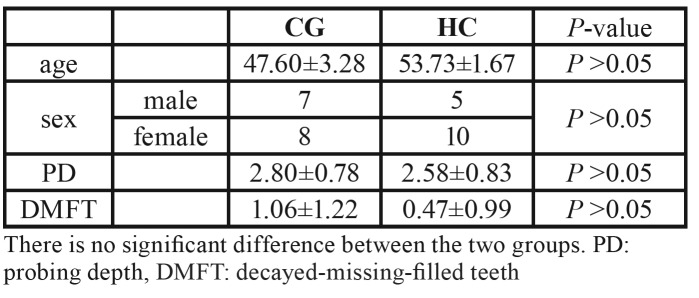
Clinical description of Cheilitis Granulomatosa patients and Healthy Controls.

In total, the number of final clean reads per sample from CG patients and HCs ranged from 10364 to 70069 and 8290 to 26444, respectively, while the OTU per sample ranged from 100 to 233 and 64 to 137, respectively (Table 2).

**Table 2:**
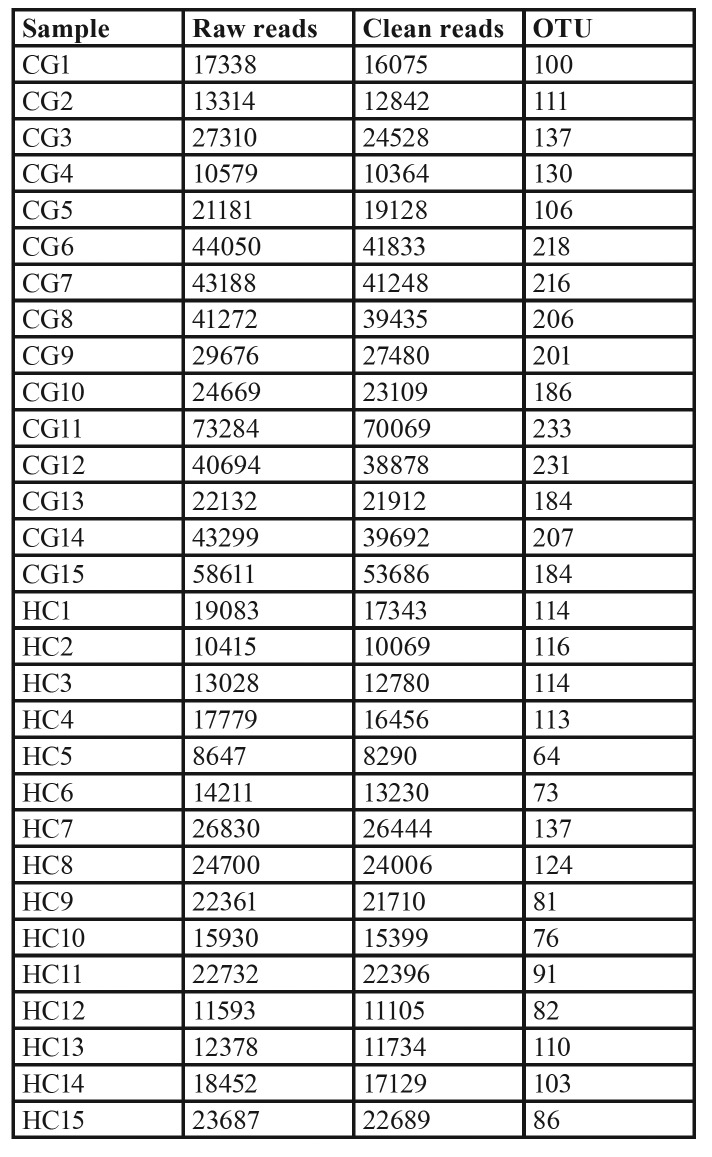
Basic sequencing information.

- CG patients had a higher microbial diversity than HC individuals

**Figure 1:**
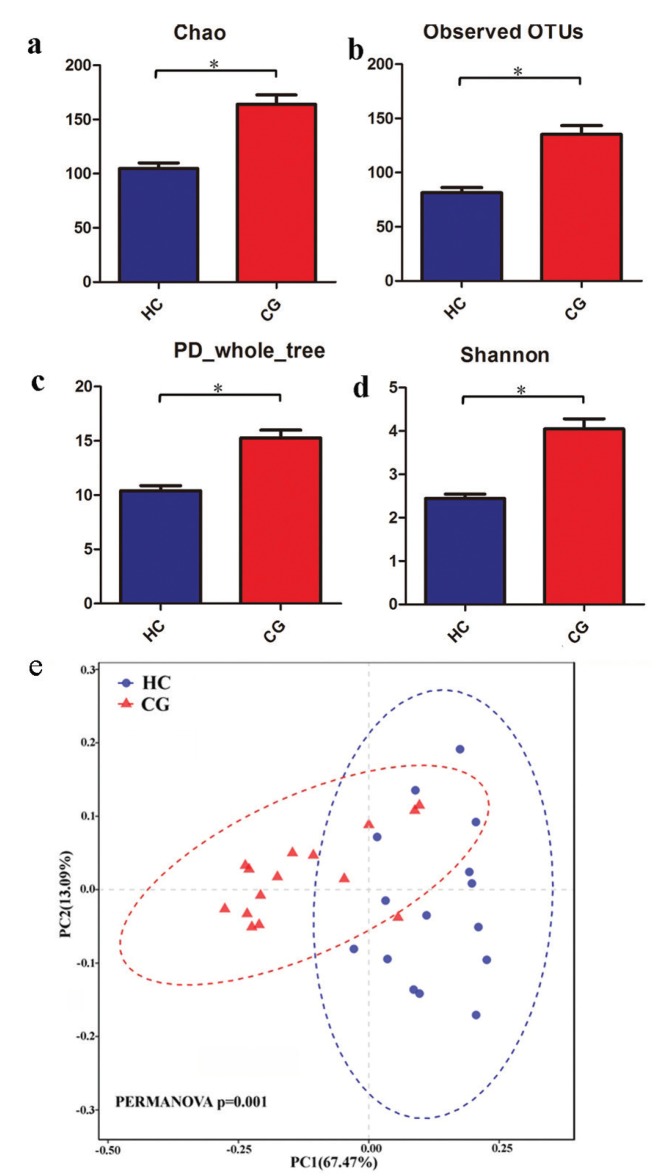
CG patients had a relative higher microbial diversity than HC individuals. 1a-1b: The Alpha diversity analysis between the two groups, which was analyzed by Kruskal-Wallis test. (* *P*<0.05); 1e. Principle co-ordinates Analysis (PCoA) analysis base on OTUs between HC and CG groups. The pairwise permutational multivariate analysis of variance (PERMANOVA), using the Adonis analysis,  was used to compared the significant difference. The HC samples were displayed in blue and CG samples were displayed in red.

Chao 1, observed OTU and PD whole tree of the CG patients were significantly higher compared with HC individuals, indicating that more bacterial richness and evenness was observed in saliva samples from CG patients compared with those from HC samples. Furthermore, CG patients had a significantly higher shannon diversity than HC individuals (Fig. 1).

Also, Principal co-ordinates Analysis depicting variable elements was detected based on OTUs information to compare the microbiota composition in those with CG and HC individuals. From Fig. 1, two elements accounted for 67.47% and 13.09%. CG patients tended to distribute apart from HC, and HC subjects had a loose distribution, while the CG clustered closely. Taken together, this result suggested that the bacterial composition and structure was significantly different between CG patients and HC (PERMANOVA *p*=0.001).

All the results indicated different salivary microbiome may occur between CG patients and the healthy.

- The core salivary microbiota in HC and CG patients

**Figure 2:**
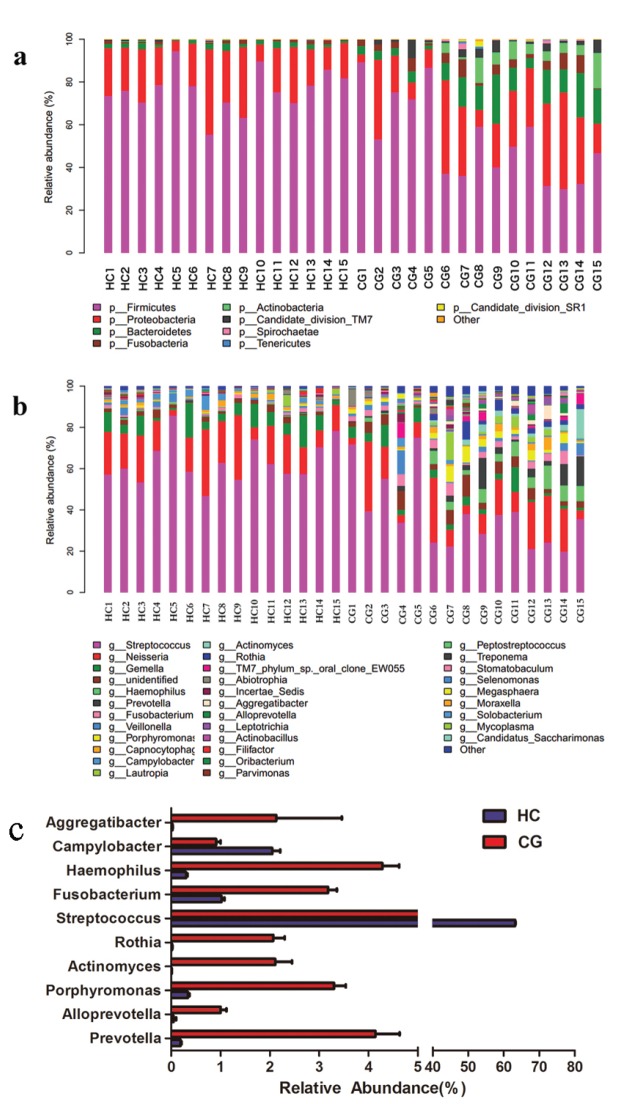
The relative abundance of bacteria communities in HC and CG patients. 1a,1b shows the main bacterial composition on phylum and genus level. 1c shows the genera with the significantly different relative abundance between HC and CG groups, which were identified by Wilcoxon signed rank test (*P* < 0.05). The bars and error bars were also calculated.

We investigated the overall microbiota composition from the phylum to the genera level in the salivary samples to see if there was a difference between HC and CG patients. Overall, there were 14 phyla, 22 classes, 39 orders, 71 families, and 84 genera identified by the pooled sequences. The results of the phylum level distribution are shown in Fig. 2.

All sequences were distributed in the oral saliva by the four phyla: Firmicutes, Proteobacteria, Bacteroidetes and Fusobacteria which accounted for 80% of all phyla. At the genus level, the most prevalent genera were Streptococcus and Neisseria, which were consistent with the phyla analysis.

Analysis at the genus level showed that 10 genera were significantly different between the two groups (Fig. 2). Prevotella, Alloprevotella, Porphyromonas, Actinomyces, Rothia, Fusobacterium, Haemophilus, and Aggregatibacter had a significantly higher relative abundance in CG patients with a mean abundance over 1%. However, Streptococcus and Campylobacter were the most abundant genera in HC individuals with a mean relative abundance of 63% and 2%, respectively. While the relative abundance of those two genera were 38% and 1% in CG patients.

- Co-occurrence network of OTUs showed different patterns between CG and HC individuals

**Figure 3:**
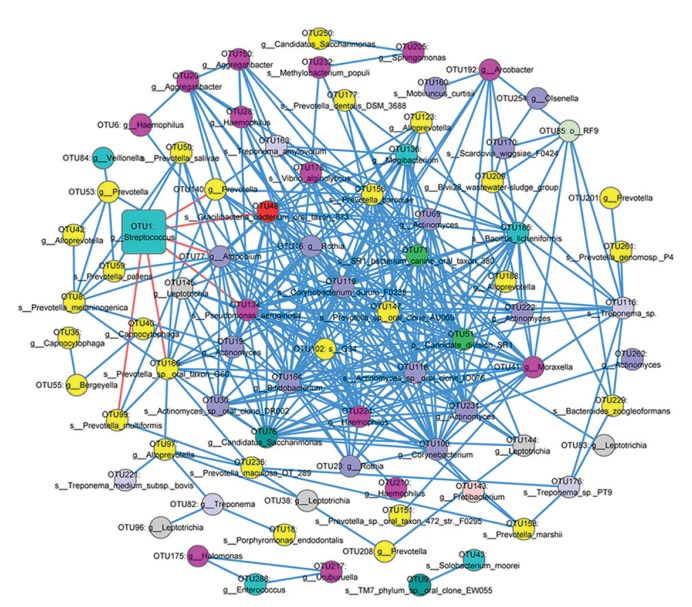
Co-occurring network of interactive bacteria between HC and CG groups. The co-occurrence network was constructed based on the sequenced OTUs with Pearson’s correlation coefficients |r| > 0.8 and Permutation test *P* < 0.01. Each node represents an OTU. HC and CG were labeled by Box and Circle, respectively and the size represented the relative abundance of OTUs. The blue and red lines indicated positive correlations and negative correlations, respectively.

Analyses at the OTU level showed that 151 OTUs were significantly different between CG patients and HC individuals. so we calculated the Pearson’s correlation coefficients for the 151 OTUs and generated a co-network between CG patients and HC individuals (Fig. 3).

There was a higher relative abundance of Streptococcus, which was consistent with the genus analysis. 325 positive and 6 negative correlations were found in samples, respectively. Most of the higher enrichment bacteria in CG patients were from the Prevotella, Actinomyces, and Rothia genera.

## Discussion

CG is a disturbing and persistent idiopathic swelling of the lip and the cause has not been wholly elucidated. Previous cases have suggested a possible relationship between CG and dental infection. It was reported that elimination of dental infectious foci resulted in regression or disappearance of swelling in 11 out of 16 patients (5). Furthermore, other cases also demonstrated that the swelling of the lip improved greatly after the treatment of apical periodontitis without any drugs (6,7). Perhaps surprisingly, Borrelia has been found in the tissue homogenates and sections of CG patients (14). The same study also found that spirochaetes tallied with the histopathological changes and noted that the swelling reduced after treatment with penicillin. These data suggested that CG was associated with infectious diseases caused by spirochaetes (15). Successful treatment of CG with roxithromycin has also been reported (16). However, these relatively few studies cannot directly or indirectly prove the finding of causative microorganisms in the tissue of CG patients. Therefore, this study focused on the salivary microbiota composition of CG patients and HCs using high-throughput sequencing methods, with the aim of comprehensively providing a new perspective in relation to the microbiota and the possible pathogenesis of CG.

In this study, four dominant phyla, Firmicutes, Proteobacteria, Bacteroidetes and Fusobacteria were detected in all selected subjects. A previous study had shown that Firmicutes, Bacteroidetes, Actinobacteria, Proteobacteria and Fusobacteria were also found in the oral cavity of healthy individuals and in those with periodontitis (8). Chloroflexi and Firmicutes have been determined as the prevailing phyla in subgingival plaques (17). These phyla may together comprise approximately 80% of oral microbiota. Perhaps surprisingly, Borrelia has been found in the tissue homogenates and sections of CG patients(14). The same study also found that spirochaetes tallied with the histopathological changes and noted that the swelling reduced after treatment with penicillin. These data suggested that CG was associated with infectious diseases caused by spirochaetes (15). The spirochaetes was found in the all the 15 CG patients, but only in 10 of the 15 healthy. There is significant difference between the two groups in the average relative abundance with 0.4% in the CG patients and 0.03% in the HC.

Our preliminary results showed that CG patients had a significantly higher microbial diversity compared with HC individuals. This illustrated that the shifts of membership that occurred in the disease group may be due to the breakdown of the oral environment between microbiota and the host (18). The implication is that any disturbance in the microbiota may be predictive of diseases (19). From the PCoA map, we can see that the disease samples were clustered tightly whereas HC samples had a loosely distribution, which means the composition of the microbiota in CG is very consistent but very different in HC patients. Inter-individual differences may lead to variation in the relative abundance and prevalence of bacteria.

In our study, there was a significant difference between HC and CG patients with respect to the following genera: Aggregatibacter, Haemophilus, Fusobacterium, Rothia, Actinomyces, Porphyromonas, Alloprevotella, Streptococcus, Campylobacter and Prevotella. The genera historically associated with periodontitis and apical periodontitis had a relative high abundance in CG patients compared with HC individuals. These included Prevotella, Alloprevotella, Porphyromonas, Actinomyces, Rothia, Fusobacterium, Haemophilus, and Aggregatibacter. Among these genera, Actinobacteria and Fusobacteria were present in most of the patients (20,21). In chronic endodontic infection, *Porphyromonas gingivalis*, Porphyromomas endodontalis, *Prevotella intermedia* and Prevotella nigrescens were observed (22) *Fusobacterium nucleatum* has been identified in high frequencies in adults affected by mild to acute periodontitis (21,23).Some species of the genus Prevotella, such as P. buccae, P. disiens, and P. oralis are often present in association with other organisms in acute endodontic infections (24), indicating that this genus may participate in the inflammatory response to infection, which also includes CG.

However, it is still unclear how the bacteria induce the granulomatous lesion of the lip. The typical pathological manifestation of CG is one or more non-caseating granulomas with epithelioid histiocytes and multinucleated giant cells and lymphocytes. The granulomatous plugs in the lymphatic system may be one of the mechanisms that cause the tissue edema (25). The proliferation and aggregations of histiocytes within the lymphatic vessels may be stimulated by a persistent antigen which was digested and presented from the microorganism (26). It is possible that there is a very abundant genus in CG patients, which has the same antigen, which induced an undesired immune reaction in CG patients. In previous research, oral pathogenic microorganisms have been found to play a role not only in infectious disease but also in tumors and other disease. Fusobacterium pathogens can invade the human host and increase virulence in the oral cavity and tumor tissues (27). Periodontal pathogens may stimulate tumorigenesis via direct interaction with cancerous and pre-cancerous oral epithelial cells through Toll-like receptors(28). Further research should be performed to illuminate how the microorganisms induce the immune reaction and recruit the cells to form the granuloma. The combined effect of the immune reaction, genetic background, and individual susceptibility is likely to be important in development of disease.

In contrast, Streptococcus and Campylobacter were less prevalent in CG patients. These results illustrated that different biodiversity may occur in different conditions to response to changes in the oral environment. One possible reason is that the high diversity and abundance of the dominant genus in CG patients changes the constitution and balance of the microbiome. It is possible that CG may have a correlation with odontogenic pathogenic microorganisms. The microbial discrepancy could be explained by the change in biomass structure.

In conclusion our preliminary findings demonstrated that CG patients had a significantly different microbial diversity and composition, which might be helpful in understanding the etiology, diagnosis and treatment of CG. But there were also same limitations in our study. 1) The sample size was relative small and no data of microbiological shift before and after the treatment can be obtained. 2) The large amplified products (468bp) raises sequence error as well as downstream bioinformatics challenges in terms of making contigs, so increase the sequence depth is necessary. 3) The underlying mechanism of how the causative microorganism(s) induced the immune reaction could be meaningful research aspects for further elaboration.
